# Transcriptome Analysis of *T. asperellum* GDFS 1009 Revealed the Role of *MUP1* Gene on the Methionine-Based Induction of Morphogenesis and Biological Control Activity

**DOI:** 10.3390/jof9020215

**Published:** 2023-02-06

**Authors:** Valliappan Karuppiah, Cheng Zhang, Tong Liu, Yi Li, Jie Chen

**Affiliations:** 1 School of Agriculture and Biology, Shanghai Jiao Tong University, Shanghai 200240, China; 2 State Key Laboratory of Microbial Metabolism, Shanghai Jiao Tong University, Shanghai 200240, China; 3 Key Laboratory of Genetics and Germplasm Innovation of Tropical Special Forest Trees and Ornamental Plants, Ministry of Education, Hainan University, Haikou 570228, China; 4 Shanghai Dajing Biotec. Ltd., Shanghai 201100, China

**Keywords:** *Trichoderma*, morphogenesis, sporulation, methionine permease, biological control

## Abstract

*Trichoderma* spp. are biological control agents extensively used against various plant pathogens. However, the key genes shared for the growth, development and biological activity are unclear. In this study, we explored the genes responsible for the growth and development of *T. asperellum* GDFS 1009 under liquid-shaking culture compared to solid-surface culture. Transcriptome analysis revealed 2744 differentially expressed genes, and RT-qPCR validation showed that the high-affinity methionine permease *MUP1* was the key gene for growth under different media. Deletion of the *MUP1* inhibited the transport of amino acids, especially methionine, thereby inhibiting mycelial growth and sporulation, whereas inhibition could be mitigated by adding methionine metabolites such as SAM, spermidine and spermine. The *MUP1* gene responsible for the methionine-dependent growth of *T. asperellum* was confirmed to be promoted through the PKA pathway but not the MAPK pathway. Furthermore, the *MUP1* gene also increased the mycoparasitic activity of *T. asperellum* against *Fusarium graminearum*. Greenhouse experiments revealed that *MUP1* strengthens the *Trichoderma*-induced crop growth promotion effect and SA-induced pathogen defense potential in maize. Our study highlights the effect of the *MUP1* gene on growth and morphological differentiation and its importance for the agricultural application of *Trichoderma* against plant diseases.

## 1. Introduction

*Trichoderma* has the function of antagonizing phyto-pathogens and promoting plant growth. Therefore, it has been widely developed as a biological control agent in agriculture. During the growth periods of *Trichoderma*, it differentiates into three different morphologies: mycelium, conidiospores and chlamydospores. Mycelium is used for rapid growth to provide maximum access to available nutrients. Two different types of propagule spores were generated due to different environmental factors, such as ROS, nutrient starvation and light [1]. Conidiospore is a dormant propagule of *Trichoderma* in which the metabolism is inactive. In the process of spore formation, the mycelial cells dehydrate themselves and do not cooperate with the germination-required enzymes [2]. Chlamydospores are produced inside or on the tip of the mycelium with a strong cell wall and compressed cytoplasm, providing a longer survival time and stronger plant disease control ability than conidiospores [3]. Morphological differentiation is an important factor not only for the propagule production and stress resistance but also for the mycoparasitic and biocontrol activity of the *Trichoderma*. However, the mechanism and the genes behind the morphological differentiation need to be clarified and require more studies.

The morphological differentiation of hyphae into chlamydospores involves complicated biological processes and pathways; its different molecular pathways and networks have been studied in yeast through RNA sequencing techniques [4,5,6]. During this process, TOR, mitogen-activated protein kinase (MAPK) pathway, and cAMP-mediated signaling pathway are necessary for cell wall integrity and amino acid sensing [7]. Amino acids are the building blocks of all proteins and are important for several cellular functions, which have been shown to be involved in the morphogenesis of *Candida albicans.* [8]. Methionine is an essential amino acid that activates the PKA pathway for yeast morphogenesis through cAMP with the help of G-protein coupled receptor (GPCR) Gpr1 [8]. In addition, methionine is a precursor of S-adenosylmethionine (SAM), further as an auxiliary substrate for polyamine formation, a biologically active substance that regulates cell differentiation. Exogenous methionine and S-adenosylmethionine regulate the synthesis of sulfate permease [9]. In *Hypocrea jecorina* (anamorph *Trichoderma reesei*), the addition of methionine in the dark enhances the transcriptional level of cellulase cbh1 [10]. Therefore, methionine transport is an important process required for the methionine-induced morphogenesis [11,12]. The membrane proteins, specifically permease, transport the amino acids from the extracellular to intracellular through the plasma membrane. Methionine is transported across the membrane to yeast cells by three different permeases, one-high affinity (*MUP1*) and two low-affinity permeases (*MUP2* and *MUP3*). The *MUP1* gene has been shown to encode an integral membrane protein with 13 putative transmembrane regions [13]. Methionine permease Mup1 is involved in methionine transport, conversion of methionine to SAM, and morphogenesis based on the cAMP-PKA pathway in *Candida albicans*. Lack of Mup1 reduced the methionine transport rate and significantly abolished filamentous structures’ formation while reintroducing this gene restored morphogenesis [8]. However, the possible mechanism and the key regulatory genes involved in the morphological differentiation in *Trichoderma* need to be clarified. 

The present study evaluated the transcriptomes of *T. asperellum* GDFS 1009 under solid-surface culture and liquid-shaking culture. Candidate genes potentially regulating growth and morphological differentiation were identified and validated. The biological function of the *MUP1* gene, a key gene encoding a high-affinity methionine permease not previously studied in *T. asperellum* GDFS 1009, was analyzed by genetic, biochemical and greenhouse plant growth assays for growth and biological control ability.

## 2. Materials and Methods

### 2.1. T. asperellum GDFS 1009 Fermentation Culture

*T. asperellum* GDFS 1009 (CGMCC NO. 9512) was cultured on potato dextrose agar (PDA) medium and provided by our laboratory. The *T. asperellum* GDFS 1009 spores were collected and prepared for a suspension with a concentration of 1 × 10^6^ spores/mL. In total, 200 μL of the spore suspension was inoculated into 200 mL potato dextrose broth (PDB) at 120 rpm in an Erlenmeyer flask for liquid shaking culture. Coated inoculation into PDA plates was completed for solid-surface culture. Both ferments were carried out in a constant temperature incubator at 28 °C with alternating light and dark for 12 h. Three sampling points with three biological replicates at 48, 72 and 96 h grown in PDA represented by the labels CN1, CN2 and CN3 and PDB represented by CH1, CH2 and CH3, respectively, were used for the RNA extraction, transcriptome sequencing, and morphological differentiation analysis.

### 2.2. RNA Isolation, cDNA Library Preparation and Sequencing

Mycelium and spores on the surface of the PDA samples were carefully scraped and collected using a sterilized microslide. All cultures in PDB samples (mycelium and chlamydospores) were collected using sterile filter paper, washed three times with sterile water, and the remaining water was removed with sterile filter paper. All samples were frozen in liquid nitrogen and used for transcriptome sequencing. RNA was extracted from the frozen samples using the FastPure Plant RNA isolation kit (Vazyme Biotech Co., Ltd., Nanjing, China), according to the manufacturer’s instructions. The RNA samples were used to prepare barcoded RNA-seq libraries using the TruSeq™ RNA sample preparation kit (Illumina, CA, USA). RNA-seq library construction and sequencing were performed in personalbio, China. Barcoded libraries were sequenced using Illumina HiSeq2000 sequencer to produce 100 bp paired-end reads. The poor-quality sequences, adapter k-mers and bases were trimmed using Trimmomatic (version 0.30, http://www.usadellab.org/cms/?page=trimmomatic, accessed on 30 May 2020) [14]. Sequence lengths of fewer than 25 nucleotides were eliminated. Filtered reads were compared with the *Trichoderma asperellum* CBS 433.97 v1.0 (https://mycocosm.jgi.doe.gov/Trias1/Trias1.home.html, accessed on 30 May 2020) using TopHat 2.0.8 release. The expression level of differentially expressed genes was calculated using SOTA and cluster analysis [15]. *T. asperellum* grown in PDA was used as a control to detect the level of gene expression of *T. asperellum* grown in PDB. The significant gene expression level was calculated using Benjamini–Hochberg multiple tests (*p* < 0.05). The upregulation and downregulation of differentially expressed genes were identified by the cuffdiff program. The functions of identified DEGs were then investigated by pathway enrichment analysis.

### 2.3. Validation of Transcriptome Data by Reverse Transcription Quantitative PCR (RT-qPCR)

Thirteen different genes of *T. asperellum* GDFS 1009 were selected to quantify its expression level under different conditions using reverse transcription quantitative PCR to validate the results of the transcriptomics study. Primers for each gene were designed using the Primer-BLAST and listed in Table S1. The specificity of the primer binding was checked using the thermal cycler with the following conditions: 94 °C for 3 min, 30 cycles of 94 °C for 30 s, 55 °C for 30 s, and 72 °C for 20 s, and a final step at 72 °C for 10 min. Total RNA was extracted from *T. asperellum* cultured for 96 h hour in PDA, PDB, M1 (KH_2_PO_4_-2 g, MgSO_4_-1 g, CaCl_2_-1 g, (NH_4_)_2_SO_4_-3 g, peptone-5 g/L), M2 (peptone-2 g; glucose-10 g/L) and YMB medium (yeast extract-20 g; molasses-20 g/L), which were purchased as raw material from Shaoxin Biotechnology Co., Shanghai, China. 

cDNA was synthesized using HiScript IIIRT supermix for qPCR (+gDNA wiper) (Vazyme, Nanjing, China) according to the manufacturer’s instructions. The synthesized cDNA was diluted with 80 μL of water and used as a template for real-time PCR using 2×ChemQ Universal SYBR qPCR Master mix (Vazyme, China). PCR program was: 94 °C for 3 min, 40 cycles of 94 °C for 30 s, 55 °C for 30 s, and 72 °C for 20 s. The expression fold was measured using the ΔΔCT method relative to the *T. asperellum* grown in PDA. Elongation factor 1 was used to normalize the gene expression. The results were the average of three independent biological replicates with qPCR technical triplicates.

### 2.4. Construction of MUP1 Gene Knockout and Overexpression Strains

The deletion of the *MUP1* gene in *T. asperellum* GDFS 1009 was performed based on the homologous recombination of a previous study [16]. An *MUP1* overexpression cassette comprising the *TrpC* promoter, *MUP1* ORF and *TrpC* terminator was cloned into the pC1300N. *Agrobacterium tumefaciens* (AGL1 Agrobacterium strain, preserved in our laboratory)-mediated transformation (ATMT) was carried out to transfer the recombinant plasmids into *T. asperellum.* Two strains were named Δ*Mup1* and OE*Mup1*, respectively. PCR and RT-qPCR were used to check the knockout and overexpression of the *MUP1* gene. The primers used in the construction of gene knockout and over-expression strains are listed in Table S2.

### 2.5. Growth and Morphology of the TAWT, OEMup1–4, OEMup1–5, ΔMup1–1 and ΔMup1–10

The growth ability of TAWT, OE*Mup1–4*, OE*Mup1–5*, Δ*Mup1–1* and Δ*Mup1–10* was tested on PDA, YMA, Vogel minimal salt medium (VMS) without ammonium sulfate (VMS/N−) and with 100 mM methionine supplement (VMS/+Met). Strains were inoculated at the center of the Petri dishes and incubated at 28 °C for 5 days. The radial growth of each strain was measured on the 3rd, 4th and 5th day. The sporulation ability of wild and mutant strains was estimated by inoculating spore suspension (1 × 10^6^/mL) in liquid media as shown above, incubating at 28 °C for 5 days, and counting by a hemocytometer.

To observe the morphology of wild-type and mutant strains, 1 mL of culture grown in PDB for 5 days was centrifuged, washed with sterile PBS and fixed with 2.5% glutaraldehyde for 1 h. The samples were post-fixed with 1% osmium tetroxide, dehydrated with acetone series embedded with epoxy, and polymerized at 55 °C for 24 h. Ultrathin sections were coated into the copper grid and visualized under a biological transmission electron microscope (Tecnai G2, FEI Company, Hillsboro, OR, USA). 

### 2.6. cAMP Measurements 

TAWT, OE*Mup1–4*, OE*Mup1–5*, Δ*Mup1–1* and Δ*Mup1–10* were pre-cultured in PDB medium at 28 °C for 2 days, and then they were centrifuged and washed with phosphate buffer (pH = 6) and incubated in PDB medium containing L-methionine, SAM and spermidine at 28 °C for 3 h. The samples were collected every 60 s after induction until 420 s. Each sample was collected and transferred into 60% methanol at 100 °C to stop the enzymatic reactions. The cAMP concentration of each sample was measured using the plant cyclic adenosine monophosphate (cAMP) ELISA Kit (Shanghai Cablebridge Biotechnology Co., Ltd., Shanghai, China).

### 2.7. RT-qPCR Analysis of Gene Expression Related to Sporulation and Mycoparasitism 

The expression of the genes related to sporulation, mycoparasitism and secondary metabolism were analyzed in the TAWT, OE*Mup1–5*, OE*Mup1–6,* Δ*Mup1–1* and Δ*Mup1–10* grown in the potato dextrose medium for 4 days. The total RNA was extracted, and cDNA was synthesized using HiScript IIIRT supermix for qPCR (+gDNA wiper) (VAZYME, Nanjing, China) and 2× ChemQ Universal SYBR qPCR Master mix (VAZYME, China), respectively. Secondary-metabolism-related genes (three NRPSs, two PKSs, O-methyl transferase B, and cytochrome P450) were measured [17]. Real-time PCR was performed according to the previous study, and the list of primers is shown in Table S3 [18]. The expression fold changes were calculated using ΔΔCT compared to the TAWT. The elongation factor 1 gene was used to normalize the gene expression. 

### 2.8. Mycoparasitism Assays 

Dual confrontations between TAWT, OE*Mup1–5*, OE*Mup1–6*, Δ*Mup1–1* and Δ*Mup1–10* and the pathogens (*Fusarium graminearum*) were carried out as previously described method [19]. A 5 mm diameter of the agar plug of TAWT, OE*Mup1–5*, OE*Mup1–6*, Δ*Mup1–1* and Δ*Mup1–10* and the pathogens (*F. graminearum*) was inoculated on either side of the PDA agar and incubated for 5 days at 28 °C. Single culture of TAWT, OE*Mup1–5*, OE*Mup1–6*, Δ*Mup1–1*, Δ*Mup1–10* and *F. graminearum* was used as a control. The percentage growth inhibition of the pathogens (*F. graminearum*) was calculated as in the previous study [19].

The expression of secondary metabolites genes related to mycoparasitism in TAWT, OE*Mup1–5*, OE*Mup1–6*, Δ*Mup1–1*, and Δ*Mup1–10* was studied using a mock antagonism assay [19]. Briefly, the TAWT, OE*Mup1–5*, OE*Mup1–6*, Δ*Mup1–1*, Δ*Mup1–10* was prepared in PDB medium, washed and transferred into 4-days-old *F. graminearum* (wheat head blight and root rot pathogen) culture grown in the PDB medium. After 24 h incubation with shaking, the cultures were harvested, frozen, and the RNA was extracted. Induction of the secondary-metabolism-related genes related to mycoparasitism activity (non-ribosomal peptide synthetase (NP1 and NP2), Putative ferrichrome synthetase (NP3), Cytochrome P450 (Tri 13) 1, O-methyl transferase (OMT) and Polyketide synthetase (PK1 and PK2)) was assessed by quantitative RT-PCR. The results were the average of three independent biological replicates with qPCR technical triplicates.

The culture filtrate was used as the crude protein extract to determine the enzyme activity. Chitinase activity was assessed using a chitinase enzyme Activity Determination Kit (Shanghai Cablebridge Biotechnology Co., Ltd., Shanghai, China). Activities of β-1,3-glucanase and cellulase were measured using a β-1,3-glucanase and cellulase enzyme Activity Determination Kit (Shanghai Cablebridge Biotechnology Co., Ltd., Shanghai, China), respectively, according to the manufacturer’s instruction. 

### 2.9. Biocontrol Assays 

The effect of the *MUP1* gene of *T. asperellum* GDFS 1009 on the plant growth and *F.graminearum* root rot control was determined by potting trials of corn (*Zea mays* L.) under greenhouse conditions. Five corn seeds were planted per pot, regularly irrigated, and kept in light at 28 °C for 12 h and darkness at 24 °C for 12 h. After 2 weeks, the seedlings were thinned to two plants per pot. After thinning, the plants were treated as follows: (T1) TAWT; (T2) OE*Mup1–5*; (T3) OE*Mup1–6*; (T4) Δ*Mup1–1*; (T5) Δ*Mup1–10*; (T6) TAWT+FG (challenged with *F. graminearum*); (T7) OE*Mup1–5*+FG; (T8) OE*Mup1–6*+FG; (T9) Δ*Mup1–1*+FG; (T10) Δ*Mup1–10*+FG; (T11) FG; and (T12) Control. The inoculum volume of different strains was 100 mL at a concentration of 1 × 10^6^ spores/mL. *F. graminearum* was inoculated after 5 days of TAWT, OE*Mup1–5*, OE*Mup1–6*, Δ*Mup1–1* and Δ*Mup1–10* inoculation. A total of 10 plants were used for each treatment, divided into 5 pots for the experiment. The maize plant growth was investigated as in a previous study, and the plant incidence was calculated as the number of deaths/total number of plants [18].

### 2.10. Statistical Analysis 

All these experiments were carried out using three replicates with at least two replications and reproducible results. The figures were plotted using Microsoft Office Excel and origin 6.0 with standard error bars. For multiple comparisons, two-way ANOVA with post hoc LSD, Duncan and Bonferroni were performed using the SPSS 2.0. Student’s *t*-test to analyze the gene expression between wild-type and mutant strains. *p* < 0.05 was considered significant.

## 3. Results

### 3.1. Morphological Growth Differences of T. asperellum GDFS 1009 in Solid-Surface Culture and Liquid-Shaking Culture

*T. asperellum* GDFS 1009 was cultivated in solid and liquid media to identify the growth kinetics of conidiospore and chlamydospore development. The mycelium of *T. asperellum* GDFS 1009 was initiated to grow in the solid medium after 24 h. Green pigmentation started at the 48th hour of incubation in the PDA and gradually increased until the fifth day. Correspondingly, conidiophores, structures carrying conidia, also began germinating after 48 h of incubation (Figure S1). More conidiospores were formed by solid-surface culture in PDA and germinated over the conidiophore after 72 h. Subsequently, the conidiospores have been detached from the conidiophore and spread throughout the medium. In contrast, more chlamydospores were formed in PDB under an incubator shaker. These chlamydospores started germinating 48 h after inoculation and gradually increased until 120 h (Table 1). As conidia and chlamydospores germinated profusely after 48 h of incubation, we selected samples of *T. asperellum* GDFS 1009 from the 48th, 72nd and 96th hours to analyze transcriptome expression under solid-surface culture and liquid-shaking culture.

### 3.2. RNA Sequencing and De Novo Assembling of T. asperellum GDFS 1009 Transcriptome

To identify the essential genes and regulatory pathways associated with chlamydospore formation, RNA sequencing of *T. asperellum* GDFS 1009 was performed at different stages under solid-surface and liquid-shaking culture conditions. After removing low-quality reads, adaptor sequences, empty reads and rRNA reads, 267,176,986 clean reads were obtained. A total of 40,076,547,900 bp were generated through the Illumina sequencing. After de novo assembling all the clean reads, a total of 12,557 contigs were generated (Table S4).

### 3.3. Differential Expression Genes of Liquid-Shaking Culture and Solid-Surface Culture 

**Figure 1:**
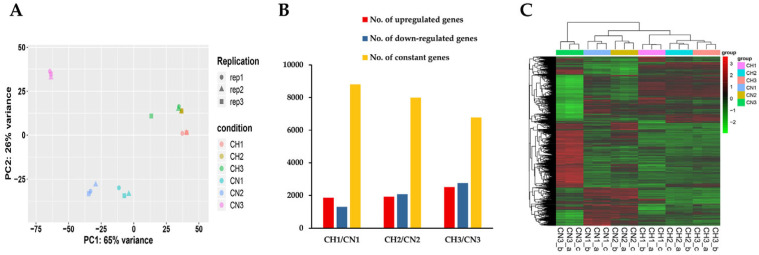
RNA expression differences of *T. asperellum* GDFS 1009 in liquid shaking culture and solid surface culture at different growth stages. CH1/2/3 and CN1/2/3 represent liquid shaking culture and solid surface culture at 48, 72, and 96 h, respectively. (**A**) Principal component analysis (PCA) of different samples. (**B**) Differentially expressed genes (DEGs) numbers of liquid shaking culture compared to solid surface culture. (**C**) Heat map of all the DEGs and the complete linkage method was used for cluster analysis.

RNA expression-based principal component analysis (PCA) showed a significant difference in the separation between CN and CH samples, suggesting a significant difference in gene expression between solid-surface and liquid-shaking culture (Figure 1A). Differentially expressed genes (DEGs) were identified by comparing liquid-shaking culture to solid-surface culture at different stages (Figure 1B). The analysis showed that 1305, 2081, and 2744 genes were up-regulated at 48 h, 72 h, and 96 h, respectively, while 1866, 1922, and 2507 genes were down-regulated in liquid-shaking culture compared to solid-surface culture. DEG clustering analysis showed that gene expression patterns of replicate samples were similar at each time but significantly different between liquid-shaking culture and solid-surface culture, especially CN3 and CH3 (Figure 1C). 

**Figure 2:**
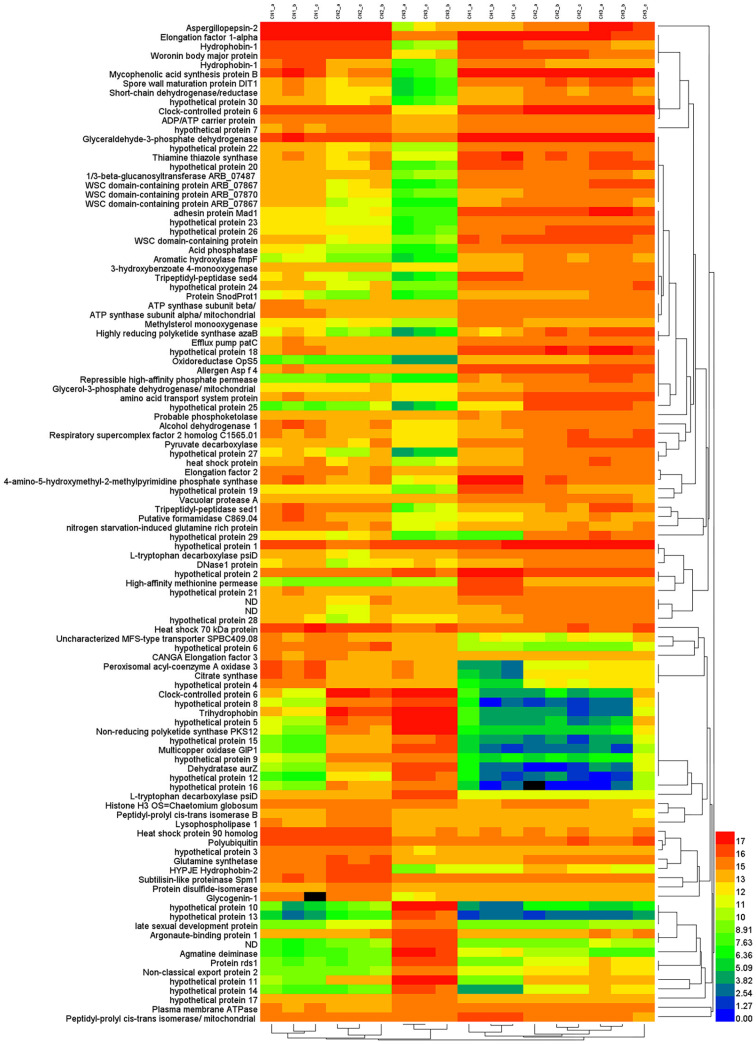
Heat map of the major DEGs between liquid-shaking culture and solid-surface culture. CH1/2/3 and CN1/2/3 represent liquid shaking culture and solid surface culture at 48, 72, and 96 h, respectively.

Several major DEGs between liquid-shaking culture and solid-surface culture are shown in Figure 2. Among them, the induction of genes associated with 1/3 beta-glucosyltransferase, WSC-domain-containing protein, acid phosphatase, dipeptidyl-peptidase sed4, methylsterol monooxygenase, highly reducing polyketide synthase azab, oxidoreductase, high-affinity phosphate permease, tripeptidyl peptidase Sed1, amino acid transport protein, and high-affinity methionine permease were upregulated. Genes such as trihydrophobin, non-reducing polyketide synthase PKS12, multicopper oxidase and dehydratase AurZ were downregulated.

### 3.4. Gene Expression Analysis and qRT-PCR Validation

Thirteen unique genes with potential roles in morphogenesis during *T. asperellum* GDFS 1009 liquid-shaking culture were chosen to validate the reliability of the transcriptome analysis by qRT-PCR. The result showed that gene expression patterns were consistent with the patterns of RNA-seq analysis, indicating that the gene expression level detected in the transcriptome is reliable (Table 2). In addition, the expression of these 13 unique genes has been analyzed on three different media (M1, M2, YM) in the same culture conditions. The RNA was extracted on the 96th hour, and its gene expression was compared with the CN3 Vs CH3 gene expression fold changes of real-time PCR and transcriptomics data. The results revealed that the expression of high-affinity methionine permease Mup1 was similar to the potato dextrose broth. Hence, the role of the *MUP1* gene was further investigated.

### 3.5. The High-Affinity Methionine Permease MUP1 Is Required for Morphogenesis

**Figure 3:**
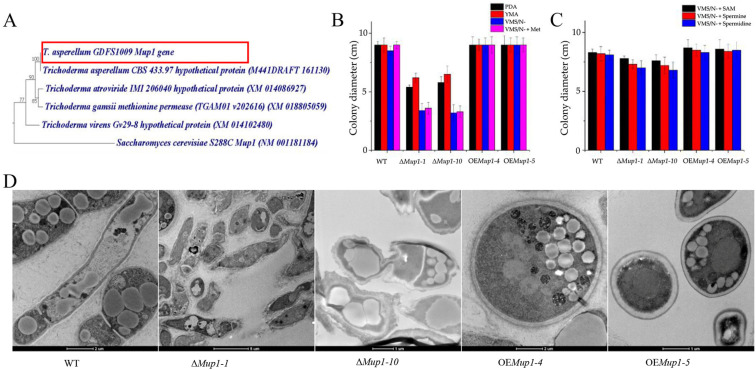
Phylogenetic tree analysis of *MUP1* and its role in the growth and morphogenesis of *T. asperellum* GDFS 1009. (**A**) Dendrogram of the *MUP1* gene family proteins. (**B**) Growth rate of WT and mutants in the different mediums with and without nitrogen source. (**C**) Growth of WT and mutants in the medium supplemented with the methionine metabolites. (**D**) Transmission electron microscopy morphology of WT and mutants, where Δ*Mup1–1*, Δ*Mup1–10* exhibit mycelial morphology due to the absence of sporulation, whereas OE*Mup1–4* and OE*Mup1–5* exhibit cell wall integrity of spores. PDA, potato dextrose agar; YMA, yeast extract molasses agar; VMS/N−, Vogel minimal salt medium (VMS) without ammonium sulfate (VMS/N−).

The ORF of *MUP1* consists of 2361 bp and encodes a 786 amino acid protein. The phylogenetic analysis showed that the *T. asperellum* GDFS 1009 *MUP1* gene was orthologous to *T. asperellum* CBS 433.97 and distantly related to the *Saccharomyces cerevisiae* (Figure 3A). To examine the significant part of the *MUP1* gene in the *T. asperellum* growth and sporulation, the overexpression cassette with the strong promoter and *TrpC*, *MUP1* and *TrpC* terminator were cloned into pCAMBIA1300 and transferred to *T. asperellum* GDFS 1009 using the ATMT method. The *MUP1* gene was knocked out using homologous recombination and ATMT. A total of 31 *MUP1* gene overexpression strains and 15 *MUP1* knockout strains were obtained. OE*Mup1–4*, OE*Mup1–5*, Δ*Mup1–1* and Δ*Mup1–10* were selected for further study.

Methionine promotes filamentation and morphological changes and is an important source for synthesizing cofactor SAM required for cellular processes [8]. To investigate the important function of methionine on the morphology and biological control activity in *T. asperellum*, we determined the role of the high-affinity methionine transport gene on methionine utilization in different media (Figure 3B). The growth rate of WT, OE*Mup1–4* and OE*Mup1–5* was higher in the VMS/N− medium compared to the Δ*Mup1–1* and Δ*Mup1–10*. Even the addition of methionine (VMS/N− +Met) failed to increase the growth rate of the *MUP1* deletion strain. Strikingly, the growth of Δ*Mup1–1* and Δ*Mup1–10* was also affected in the PDA, implying that the deletion of *MUP1* reduces the mycelial growth rate by limiting the transport of other amino acids even with adequate nutrient supply. The effect of the *MUP1* gene on the sporulation of *T. asperellum* was tested on different media, including PDB, YMB, and VMS with or without 100 mM methionine (Table 3). The absence of Mup1 visibly eradicated the induction of sporulation, whereas the overexpression of the *MUP1* gene induced the sporulation in PDB and VMS with and without a nitrogen source. A lower sporulation was observed in the Mup1 mutant on the YMB medium. This could be due to a mixture of amino acids in molasses and cornmeal. 

We observed rapid changes in the cell surface of the *MUP1* gene deletion strain, which may be associated with altered cell-wall morphogenesis. Hence, the role of the *MUP1* gene on the cell wall integrity of *T. asperellum* GDFS 1009 was observed by transmission electron microscopy (TEM). The longitudinal and cross sections of WT, Δ*Mup1–1*, Δ*Mup1–10*, OE*Mup1–4* and OE*Mup1–5* hyphae grown in PDB exhibited cell wall thickness change (Figure 3D). The cell walls of OE*Mup1–4* and OE*Mup1–5* were the most intact and the thickest (>0.2 μm), followed by the cell walls of WT. In contrast, Δ*Mup1–1* and Δ*Mup1–10* cell walls were the thinnest (<0.2 μm) and exhibited breakdown.

To evaluate whether Mup1 is required for methionine metabolism and *T. asperellum* growth, we analyzed the growth of *T. asperellum* with D-methionine, which was not utilized or metabolized by fungus. L-methionine and D-methionine showed promotion and inhibition of sporulation in WT, OE*Mup1–4* and OE*Mup1–5*, respectively. In addition, L-methionine utilization can be competitively inhibited by D-methionine in a medium supplemented with equal amounts of both methionines. The results revealed that the *MUP1* gene plays an important role in morphological sporulation through methionine metabolism. Therefore, growth and sporulation of *T. asperellum* were tested in media supplemented with methionine metabolites important for fungal growth to further explore the relationship between the high-affinity methionine permease Mup1 and methionine metabolites. Three important methionine metabolites, namely SAM, spermidine and spermine, could induce the growth of WT, Δ*Mup1–1*, Δ*Mup1–10*, OE*Mup1–4* and OE*Mup1–5* (Figure 3C). The results indicated that the metabolites could be directly absorbed by *T. asperellum* to promote growth independent of the *MUP1* gene.

### 3.6. The Role of Mup1 in the Methionine-Induced PKA Signaling Pathway

**Figure 4:**
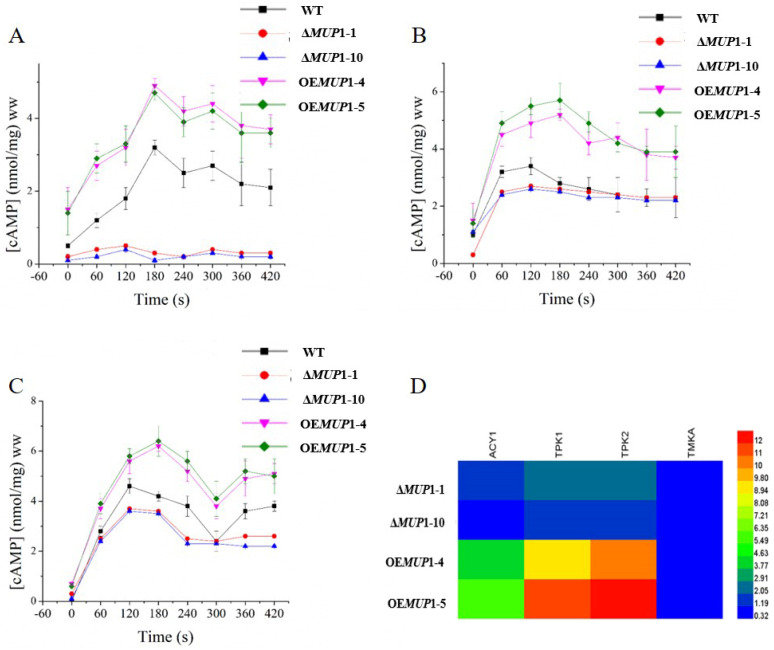
Methionine-dependent cAMP production depends on Mup1 and can be induced by SAM and spermine under the same conditions. cAMP production in response to (**A**) methionine, (**B**) SAM and (**C**) Spermine. The ww on the y-axis is the wet weight. (**D**) Expression of MAPK and cAMP signaling pathway genes in mutant strains compared to WT.

Methionine-induced growth of fungus is mediated through the PKA signaling pathway [20]. Hence, we estimated the cAMP content of WT, Δ*Mup1–1*, Δ*Mup1–10*, OE*Mup1–4* and OE*Mup1–5* grown in the low-glucose-containing VMS medium supplemented with methionine. The supplementation of methionine improved the cAMP content of WT immediately after one minute of adding WT in the medium (Figure 4A). Remarkably, the cAMP was reliant on the Mup1, and subsequently, the cAMP content was not increased in the Δ*Mup1–1* and Δ*Mup1–10* strains upon the supplementation of methionine. In addition, the cAMP levels were highly increased by the overexpression of the *MUP1* gene. Further, we analyzed the role of methionine metabolites such as SAM and spermine on cAMP production. Adding methionine metabolites such as SAM and spermine improved the cAMP content of Δ*Mup1–1* and Δ*Mup1–10* compared to the methionine (Figure 4B,C). The results showed the rapid production of cAMP by metabolites such as SAM and polyamine, independent of the *MUP1* gene. Therefore, Mup1 increases SAM production by facilitating the conversion of methionine into metabolites. The role of the PKA signaling pathway on the methionine-induced growth of *T. asperellum* was studied. The expression of *TPK1*, *TPK2* and *ACY1* genes was downregulated by the deletion of *MUP1* gene (Figure 4D). The expression of the *TMKA* gene involved in the MAPK signaling pathway was not expressed in Δ*Mup1–1*, Δ*Mup1–10*, OE*Mup1–4* and OE*Mup1–5*. The results revealed that the methionine-dependent growth of *T. asperellum* is facilitated through the PKA pathway and not the MAPK pathway.

### 3.7. Influence of T. asperellum MUP1 Gene on Mycoparasitism Activity 

**Figure 5:**
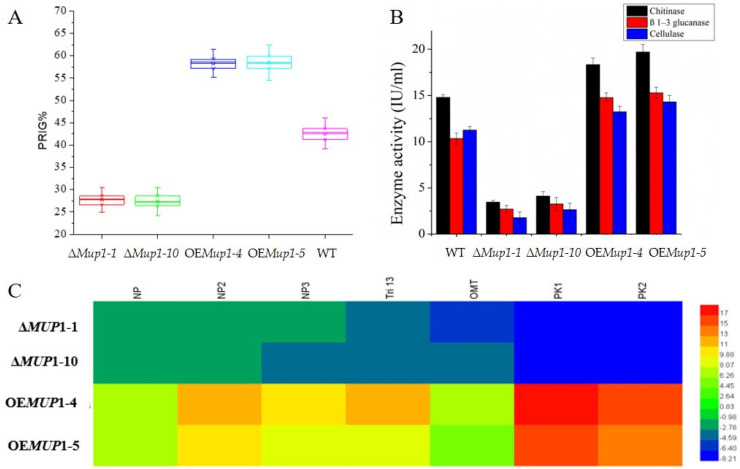
Influence of *T. asperellum MUP1* gene on mycoparasitism activity. (**A**). Percentage of inhibition of radial (PIRG) growth of *F. graminearum* by the wild type and mutants. (**B**). Enzyme activities associated with the mycoparasitism activity. (**C**). Expression of secondary metabolism related genes related to mycoparasitism activity (non-ribosomal peptide synthetase (NP1 and NP2), Putative ferrichrome synthetase (NP3), Cytochrome P450 (Tri 13) 1, O-methyl transferase (OMT) and Polyketide synthetase (PK1 and PK2).

Phytopathogen inhibition of WT and mutants was performed by plate antagonism against *F. graminearum*. The results showed that OE*Mup1–4* and OE*Mup1–5* significantly increased the percentage growth inhibition, while Δ*Mup1–1* and Δ*Mup1–10* significantly decreased the inhibition compared to WT (Figure 5A). The mycoparasitic-related enzyme activity, such as chitinase, β 1–3 glucanase and cellulase, was reduced in Δ*Mup1–1* and Δ*Mup1–10* but increased in OE*Mup1–4* and OE*Mup1–5* (Figure 5B). In addition, the role of *Mup1* on the secondary-metabolite-related genes of *T. asperellum* was studied using real-time PCR (Figure 5C). The deletion of the *MUP1* gene downregulated the expression of non-ribosomal peptide synthetase (NP1 and NP2), putative ferrichrome synthetase (NP3), cytochrome P450 (Tri 13) 1, O-methyl transferase (OMT) and polyketide synthetase (PK1 and PK2) compared with overexpression of the *MUP1* gene.

### 3.8. Influence of MUP1 Gene on the Maize Plant Growth and Biocontrol Activity

**Figure 6:**
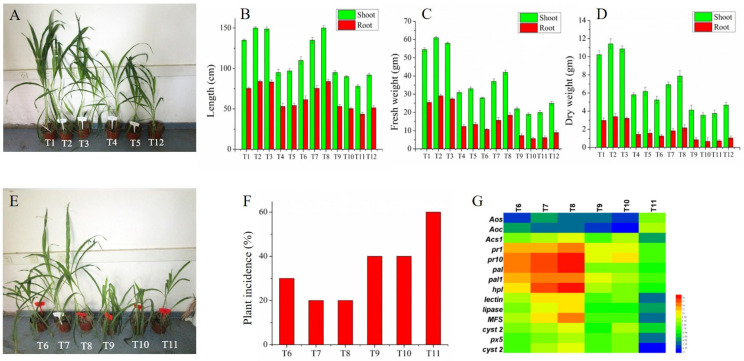
Influence of *MUP1* gene on plant growth and biological control of *F. graminearum* in the greenhouse. (**A**) Maize morphology without pathogenic stress. (**B**–**D**) Shoot and root physiological indicators under normal and biotic stress. (**E**) Maize morphology with *F. graminearum* stress. (**F**) Disease index of *F. graminearum* infected root. (**G**) Differential expression profiles of defense-related genes in maize root tissues against *F. graminearum.* (T1) TAWT; (T2) OE*Mup1–5*; (T3) OE*Mup1–6*; (T4) Δ*Mup1–1*; (T5) Δ*Mup1–10*; (T6) TAWT+FG (challenged with *F. graminearum*); (T7) OE*Mup1–5*+FG; (T8) OE*Mup1–6*+FG; (T9) Δ*Mup1–1*+FG; (T10) Δ*Mup1*–10+FG; (T11) FG; (T12) Control.

In the present study, the role of the *MUP1* gene on plant-growth-promoting and biocontrol effects against *F. graminearum* in maize root infection was studied by measuring physical characteristics and plant incidence (Figure 6). The maize plants treated with the OE*Mup1–4* and OE*Mup1–5* strains under both infected and non-infected conditions increased the plant growth parameters such as plant height and root and shoot weight compared to the control and wild-type strains (Figure 6B–D). In contrast, the knockout of the *MUP1* gene in the strains Δ*Mup1–1* and Δ*Mup1–10* reduced the plant growth compared to the wild-type strains. The inoculation of *F. graminearum* decreased the growth of the maize plants compared to the non-infected plants. 

The role of the *MUP1* gene on the defense potential of maize plants was studied by inoculating mutant and wild strains of *T. asperellum* against the *F. graminearum* in the maize roots (Figure 6E). The OE*Mup1–4* and OE*Mup1–5* (T7 and T8)-treated plants significantly reduced the disease index compared to the control (Figure 6F). In contrast, inoculated plants with Δ*Mup1–1* and Δ*Mup1–10* exhibited more disease indices than inoculated WT. This revealed that the *MUP1* gene is required for *T. asperellum* to enhance the biological control activity against the maize root rot pathogen. The defense-related gene expression of maize roots was studied (Figure 6G). The results showed that the gene expression of alkene oxide synthase (AOS) and allene oxide cyelase (AOC), enzymes related to jasmonic acid synthesis, was decreased in *T. asperellum*-treated plants. In contrast, ethylene biosynthesis key rate-limiting enzyme *Acs1* gene synthesis was increased, especially in OE*Mup1–6* treatment (T8). *PR1* and *PR10* genes involved in the systemic acquired resistance pathway (SAR) were increased by overexpression of the *MUP1* gene but reduced in plants treated with Δ*Mup1–1* and Δ*Mup1–10*. Salicylic-acid-pathway-related genes *PAL* and *PAL1* were also increasingly expressed by overexpression of the *MUP1* gene. Similarly, the HPL, lectin, lipase, MFS, Cyst2, PX5, and Cyst2 expression were also increased in the OE*Mup1–4* and OE*Mup1–5*-treated plants. The results revealed that the *MUP1* gene stimulated *T. asperellum* to improve the pathogen defense potential of plants mainly through the SA-induced SAR pathway.

## 4. Discussion

*Trichoderma* is a biological control fungus widely used to antagonize various phytopathogens [21]. Vegetative mycelium formation is essential for *Trichoderma* growth and differentiation into different types of spores. In this study, mycelium growth was observed in both media. Later the chlamydospore and conidiospore were developed from the liquid-shaking culture and solid-surface culture, respectively. This phenomenon was usually observed in the fermentation production of *Trichoderma* propagules, which may be due to nutrient stress [3]. Although the production of chlamydospore from *Trichoderma* has been studied for decades, the mechanism and key genes for chlamydospore generation still need to be clarified. Hence, we aimed to investigate the key genes for morphological differentiation of *T. asperellum* GDFS 1009 by DEG comparison of liquid-shaking culture with solid-surface culture at different time points (48, 72 and 96 h). At 48 h, the formation of vegetative mycelium was observed under both media. In the solid medium, conidiophores were produced but with fewer conidiospores, while in the liquid-shaking culture, chlamydospores started germinating. A total of 1304 genes were upregulated in liquid-shaking culture during this stage compared to solid-surface culture. This suggests that the genes related to the chlamydospores were activated in the liquid-shaking culture, while the genes involved in conidiospores might have been downregulated. The increase in the number of DEGs at 96 h indicated an increase in sporulation at this stage, with differentiation into chlamydospores or conidiospores under different conditions. The transcriptomics study showed that the group of DEGs were involved in the morphogenesis and sporulation of *Trichoderma* under liquid-shaking culture.

Among the DEGs, the cell-wall-degrading enzymes, including the chitinases and serine protease, were induced but not induced in *Clonostachys rosea* under definite environmental stimulus [22]. Several enzymes might help to dissolve the *Trichoderma* hyphae during the chlamydospore release. 1,3-Beta-glucan synthase is a glucosyltransferase enzyme involved in beta-glucan generation in fungi induced in chlamydospore germination. Glycosyltransferase has been previously reported to be involved in cell-wall remodeling in *Neurospora crassa* [23]. Glycosyltransferase gene expression was also detected during chlamydospore production of *T. harzianum* Tr-92, indicating its important role in forming the chlamydospore cell wall [24]. The tripeptydyl peptidases Sed4, SedB and SedC as exoproteases and aspartic protease Pep1 as endoproteases constitute a set of proteases in *Aspergillus fumigatus* to degrade proteins at acidic pHs and generate nitrogen sources in decomposing organic matter and composts to strengthen the cell wall of the fungus [25]. Thus, the *Sed4* upregulated in the chlamydospore may also play a role in the proper nutrition of the *Trichoderma* during the chlamydospore development. In addition, the genes involved in the membrane formation, including spore wall maturation protein DIT1, adhesion protein Mad1 and methylsterol mono oxygenase, have been upregulated during the chlamydospore production. Mutation of either DIT1 or DIT2 result in spores lacking the outermost spore wall layer [26]. Adhesion protein Mad1 at the surface of *Metarhizium anisopliae* conidia or blastospores is required to orientate the cytoskeleton and stimulate gene expression in the cell cycle. Its disruption delays germination and suppresses blastospore formation [27]. Methylsterol monooxygenase catalyzes the biosynthesis of ergosterol, which regulates membrane fluidity and permeability and maintains membrane enzyme activity, thus participating in the chlamydospore formation process [28].

Methionine is a main inducer of morphogenesis, triggering a yeast-to-hyphae switch in Lee’s medium [8]. The methionine transport-dependent protein, high-affinity methionine permease Mup1, showed upregulated expression during chlamydospore production in this study. We demonstrated that L-methionine promotes mycelial growth and sporulation of *T. asperellum* GDFS 1009, while D-methionine does not induce the growth and sporulation due to the lack of absorption by *T. asperellum*. Deletion of the *MUP1* gene inhibited methionine transport and suppressed sporulation. Although fungi can also synthesize methionine or convert cysteine to methionine via the trans-sulfuration pathway, it appears that methionine in the environment is more efficient and convenient, and therefore important for inducing asexual mycelial growth and sporulation [29]. Furthermore, using methionine is important to maintain cellular homeostasis and all biosynthetic pathway. 

Methionine is a precursor of S-adenosylmethionine (SAM), a propyl donor of polyamines such as spermine and spermidine [30]. These polyamines have been reported to regulate cellular processes such as dimorphism, spore germination, and appressorium formation and conidiation [31]. We observed that supplementation of SAM, spermidine, and spermine restored the normal growth rate of Δ*Mup1–1* and Δ*Mup1–10*. Therefore, we speculate that the *MUP1* gene may not directly regulate polyamine-mediated fungal growth but indirectly induces SAM and polyamine synthesis by transporting methionine from the external environment. Previous studies have shown that by inhibiting SAM production, the production of polyamines was stopped, and cAMP levels were blocked, thus inhibiting fungal growth, even after adding methionine [8]. Our study demonstrated similar results that Mup1 deletion completely inhibited methionine-mediated cAMP production, while in supplementation with SAM, spermine partially restored cAMP production. The signaling pathways, such as cAMP and MAPK, are interconnected and regulate the growth of the fungus [32]. In the present study, we observed that the *MUP1* gene promotes methionine transport and enhances the cAMP content, activating the PKA signaling pathway but not the MAPK signaling pathway to improve the growth of *T. asperellum.*

The morphological differentiation is important for mycoparasitism and bio-control activity [33]. Remarkably, mycoparasitism requires initial recognition of *Trichoderma* and fungal pathogen, which was involved a series of genes, some of which were related to the morphogenesis and biocontrol activity, e.g., the up-regulation of genes encoding cell-wall-degrading enzymes and secondary metabolites antagonistic to plant pathogen or stimulative to plant growth [34]. In this study, we found that deletion of the *MUP1* gene of *T. asperellum* GDFS 1009 reduced the mycoparasitic-related enzymes and secondary-metabolite-related genes, thereby reducing the antagonistic activity against *F. graminearum*. Different mechanisms are established between *Trichoderma* and plants to improve crop growth and resistance to biotic and abiotic stress [34]. The metabolites of *Trichoderma* promote communication and induce growth and biological control activity. Our study revealed that the *MUP1* gene stimulates the growth and mycoparasitism activity of *T. asperellum* GDFS 1009, thereby promoting *Trichoderma*-stimulated crop growth and disease resistance.

## 5. Conclusions

A key gene for morphological differentiation in *T. asperellum* GDFS 1009, *MUP1*, was identified by transcriptome comparison and RT-qPCR validation. *MUP1* regulates the production of SAM and polyamines involved in the PKA signaling pathway and cAMP content by controlling the transport of methionine, which affects the growth and sporulation of *Trichoderma*. Furthermore, the *MUP1* gene enhanced the growth-promoting effect of *T. asperellum* GDFS 1009 on maize seedlings and resistance to *F. graminearum.* This study confirmed the importance of the *MUP1* gene for the chlamydospore morphological differentiation and mycoparasitism function of *Trichoderma* in green agricultural production.

## Figures and Tables

**Table 1:** Conidiospore and chlamydospore formation of *T. asperellum* GDFS 1009 in different media.

Incubation Time (Hour)	Conidiospore in Solid Surface Culture (Spores/mL)	Chlamydospores in Liquid Shaking Culture (Spores/mL)
24	-	-
48	-	-
72	(9.3 ± 0.47) × 10^5^	(5 ± 0.32) × 10^3^
96	(7.2 ± 0.52) × 10^7^	(9 ± 0.58) × 10^5^
120	-	(8.5 ± 0.41) × 10^8^

**Table 2:** Comparison of 13 selected different expression genes detected by qRT-PCR and RNA-seq.

Gene Name	Real-Time PCR Data			Transcriptomics Data			Realtime PCR Data of Different Medium		
	CH1vsCN1	CH2vsCN2	CH3vsCN3	CH1vsCN1	CH2vsCN2	CH3vsCN3	M1	M2	YM
Thiamine thiazole synthase	1.0045 ± 0.12	2.3830 ± 0.76	6.3995 ± 0.35	1.72 ± 0.21	2.10 ± 0.36	4.437 ± 0.51	0.231 ± 0.078	0.229 ± 0.012	0.73 ± 0.69
Adhesin protein MAD1	11.01 ± 0.28	39.65 ± 0.53 *	113.53 ± 0.92 *	13.78 ± 0.48	23.055 ± 0.91	448.69 ± 0.27 *	0.010 ± 0.002	0.033 ± 0.01	0.06 ± 0.002
Acid phosphatases acp	6.10 ± 0.72	9.11 ± 0.87	39.43 ± 1.5 *	8.64 ± 0.95	6.15 ± 0.21	34.14 ± 1.93	0.066 ± 0.005 *	0.058 ± 0.001	0.162 ± 0.05
FAD binding domain-containing protein	1.11 ± 0.03	2.77 ± 0.18	3.82 ± 0.095	1.31 ± 0.082	1.44 ± 0.02	2.621 ± 0.043	0.115 ± 0.0093	0.084 ± 0.002	0.1 ± 0.006
glycosyltransferase	321.00 ± 0.91 *	559.94 ± 0.51 *	419.21 ± 0.72 *	266.34 ± 0.85 *	575.74 ± 0.35 *	409.46 ± 0.92 *	42.12 ± 0.85 *	68.67 ± 0.23 *	142.36 ± 0.28 *
ABC1 domain-containing protein	5.013 ± 0.07	11.037 ± 0.04	96.11 ± 0.02 *	5.21 ± 065	9.64 ± 0.19	91.852 ± 0.36 *	0.056 ± 0.02	0.004	0.0032
Urea active transporter	100.00 ± 0.47 *	122.03 ± 0.76 *	84.67 ± 0.37 *	103.21 ± 0.82 *	128.62 ± 0.56 *	87.75 ± 0.49	0.033 ± 0.001	0.108 ± 0.003 *	0.68 ± 0.006
High-affinity methionine permease	69.26 ± 0.45 *	30.884 ± 0.67 *	17.61 ± 0.71	69.51 ± 0.37 *	34.76 ± 0.28 *	17.36 ± 0.36	39.33 ± 0.42 *	87.94 ± 0.93 *	129.0 ± 0.89 *
Tripeptydyl peptidase-sedD	0.22 ± 0.006	1.18 ± 0.003	78.27 ± 0.54	0.21 ± 0.003	1.002 ± 0.004	49.74 ± 0.3	0.4585 ± 0.001	0.054 ± 0.0001	1.05 ± 0.001
Methylsterol monooxygenase	9.007 ± 0.03	8.08 ± 0.1	38.92 ± 0.6	8.93 ± 0.1	5.71 ± 0.84	33.01 ± 0.92	0.0347	0.032	0.09
Oligopeptide transporter 7	8.008 ± 0.67	8.82 ± 0.53	55.04 ± 0.98	8.41 ± 0.67	8.86 ± 0.04	47.92 ± 0.49	0.1471 ± 0.004	0.507 ± 0.008	7.87 ± 0.02
Repressible high-affinity phosphate permease	55.04 ± 0.84	213.90 ± 0.45 *	717.12 ± 3.6 *	52.69 ± 0.76	210.53 ± 1.45 *	707.00 ± 1.23 *	0.073	0.060	5.26 ± 0.006
Spore wall maturation protein	−4.960	−6.35	4.47	−4.76	−5.92	6.36	ND	ND	ND

M1 (KH_2_PO_4_-2 g, MgSO4-1 g, CaCl_2_-1 g, (NH_4_)_2_SO_4_-3 g, peptone-5 g/L), M2 (peptone-2 g; glucose-10 g/L) and YM medium (yeast extract- 20 g; molasses 20 g/L). Data resulted from biological triplicate cultures with qPCR technical triplicates. The values are the standard error of the mean. * Represent significant differences between the axenic and co-culture (*p* < 0.005).

**Table 3:** Chlamdydospore production of WT and mutants on different liquid medium.

STRAINS	PDB	YMB	VMS/N−	VMS/N− +LMET	VMS/N− +DMET	VMS/N− +L&DMET
WT	8.3 × 10^7^ ± 0.4	9.1 × 10^8^ ± 0.64	6.8 × 10^3^ ± 0.61	4.5 × 10^6^ ± 0.84	0.1 × 10^2^ ± 0.34	0.01 × 10^2^ ± 0.59
Δ*Mup1–1*	0	1.4 × 10^2^ ± 0.78	0	0	0	0
Δ*Mup1–10*	0	1.8 × 10^2^ ± 0.59	0	0	0	0
OE*Mup1–4*	9.2 × 10^7^ ± 0.56	8.8 × 10^9^ ± 0.85	3.4 × 10^4^ ± 0.76	5.6 × 10^7^ ± 0.75	0.2 × 10^2^ ± 0.54	0.09 × 10^2^ ± 0.78
OE*Mup1–5*	1.2 × 10^8^ ± 0.46	7.9 × 10^9^ ± 0.87	2.6 × 10^4^ ± 0.68	4.96 × 10^7^ ± 0.56	0.1 × 10^2^ ± 0.42	0.07 × 10^2^ ± 0.84

PDB, potato dextrose broth; YMB, yeast extract molasses broth; VMS/N−, Vogel minimal salt medium (VMS) without ammonium sulfate (VMS/N−); VMS/N− +L&DMet, Vogel minimal salt medium without ammonium sulfate supplemented with 100 mM L-methionine and D-methionine.

## Data Availability

The data presented in this study are available on request from the corresponding author.

## Supplementary Materials

The following supporting information can be downloaded at: https://www.mdpi.com/article/10.3390/jof9020215/s1, Figure S1: Conidiophores (CP), conidiospores (CS), chlamydospores (CD) and mycelium (M) morphology of *T. asperellum* GDFS 1009 under the light microscope at different time intervals; Figure S2: Construction of the Δ*Mup1* and OE*Mup1* mutant strain; Table S1: Sequences of the primers for 13 selected different expressions genes; Table S2: Primers used in construction of gene knockout and over-expression strains; Table S3: Sequences of the primers used for the RT-qPCR; Table S4: The summary of the RNA sequencing and assembly.
