# Supplementary material for: Transcriptome Analysis of T. asperellum GDFS 1009 Revealed the Role of MUP1 Gene on the Methionine-Based Induction of Morphogenesis and Biological Control Activity
Source: J Fungi (Basel). 2023 Feb 6;9(2):215. doi: 10.3390/jof9020215 (PMC9963050; doi:10.3390/jof9020215)
Supplement: Supplementary file 1 [file jof-09-00215-s001.zip › jof-2133764-supplementary.pdf]

Table S1. Sequences of the primers for 13 selected different expressions genes

Gene	Forward (5′-3′)	Reverse (5′-3′)	reference sequence
Thiamine thiazole synthase	CTGGCTGTTGCC CCTTATGA	GAACCATAGCGC CGAAGGTA	XM_024908 518
Adhesin protein MAD1	CCTTGGAAGCTC CTGGACTC	TTGGCGTAGATGC AGTCGAA	XM_024905 914
Acid phosphatases acp	TTAACCACGTTC CTCGTCCG	CGCTGAGTCGAGC ACAGTAT	XM_024908 241
FAD binding domain-containing protein	TTACTGGTTCTTG ACCGGCG	GGCACTTCGAAAC TGTTCGG	XM_024900 289
glycosyltransferase	ATTCAGGCCTCTT TTCTCTC	GGCGATGATAGTTT TATTTACTAC	
ABC1 domain-containing protein	AGGCGGCCATTA CATCTTCC	CTCACCGAAGAA GCCAGAGG	XM_024908 084
Urea active transporter	GATAGGATTGGG CGTTGGCT	TATCCGATGCCTT TGGGTCG	XM_024900 533
High-affinity methionine permease	TTCGGGAATGAC AGAGTGCC	ATTATGCCTCACG CCCAGAG	XM_024901 752
Tripeptydyl peptidase -sedD	GAGCAAAGGAT GGCTCGACT	TGCCAACCGTGAT GTCGTTA	XM_024900 328
Methylsterol monooxygenase	AAAAATCTCCCT TCCCGCCC	CTGTTGTTTGGCTG TGCCTC	XM_024904 940
Oligopeptide transporter 7	CGGATCCAAATG GGGGTTCA	CCACAGGTCGTAG CGGTATC	XM_024901 737
Repressible high-affinity phosphate permease	TCATGCTGGGCA TCGTCTAC	CTAACCGTGCCGG CGTATAA	XM_024907 66
Spore wall maturation protein DIT1	TCATTCAAGGCT ACGGCCAG	AAACCCCGTAGG AATGACGG	NW_02020 8848

Table S2. Primers used in construction of gene knockout and over-expression strains

Strain and primers	sequence (5'-3')	restriction site
**GDFS 1009**		
**pmup1-F**	CCCAAGCTTGGGAGAGCGCTCGAGCTACAATG	*Hin*dIII
**pmup1-R**	AACTGCAGAACCAATGCATTGGGTACGCGCTCATCAACATCG	*pst* I
**dmup1-F**	CGGGGTACCCCGCAGGCGTGGCAAATATACCG	*kpn1*
**dmup1-R**	CCGGAATTCCGGAAATTCGCAGGGAAGCGGAT	*ecor1*
**tDNA-F**	CCTCTTCGCTATTACGCC	
**hph-R**	ACATCGCCTCGCTCCAGT	
**mup1-F**	ATGGCGCAAGACGTGCCCAGTG	
**mup1-R**	CTATTCTTCAGCAAGTTGGAAGCCCT	
**pro--F**	TAAAACGACGGCCAGTGCCAGGAGGTCAACACATCAAT	pro--F
**pro-R**	TGGACGGCGTCGCCATTTGGATGCTTGGGTAGAA	pro-R
**ove-mup1-F**	TTCTACCCAAGCATCCAAAATGGCGCAAGACGTGCCCA	ove-mup1-F
**ove-mup1-R**	CACATTATTATGGAGAAATACTATTCTTCAGCAAGTTGGA	ove-mup1-R
**term-F**	GAGGGCAACATGAATGGGCTATTTCTCCATAATAATGTG	term-F
**term-R**	GACCTCCGAATTCGAGCTCGAAATTGACGCTTAGAC	term-R

.

Table S3. Sequences of the primers used for the RT-qPCR.

Gene	Forward (5′-3′)	Reverse (5′-3′)	Reference
**NP1**	GCGAATCAGAACAACAGCC	CATAGCCGTTCAGCCCATC	[18]
**NP2**	CGTCCGTGGATATCCAGGC	GCCATCCGTATAGCCTGAC	
**NP3**	CAAGACGCGTTTCACCTTCTTG	CGCTGTCCATTTGATCTCGC	
**Tri 13**	CATGGATGCAATCTGGGCCATTGT	TGGCCGCCCATAATAAATCCGAGA	
**OMT**	CACTGTTGCACAGGCTGTTCCATT	AAGTTGTACCACTGCTCCTCGGTT	
**PK1**	AAGACAATCCAACCTATCGGGCCA	TCTGCAACATCACAAGGCACAACG	
**PK2**	CGCGCAACTTCAACGCTCTTACAA	TCATAGGCACAAATACCTCCCGCA	
**18S rRNA**	GGTGGAGTGATTTGTCTG	CTTACTAGGGATTCCTCG	[18]
**AOS**	ACCTGTTCACGGGCACCTAC	CGAGGAGCGAGGAGAAGTTG	[18]
**AOC**	CCCCTTCACCAACAAGGTGT	ACCGAGATGTGGCCGTAGTC	
**ACS1**	GATGGTCTCGGATGATCACA	GTCGGGGGAAAACTGAAAAT	
**PR1**	CTGGGTGTCCGAGAAGCAGT	CGGGTTGTAGCTGCAGATGAT	
**PR10**	GTCATGCCGTTCAGCTTCAT	TGTTCTTGCACTCGACTTG	
**PAL**	AAGAAGGTGAACGAGCTGGA	GTTGTCGTTCACGGAGTTGA	
**PAL1**	TGTGCGTGCTTCTGCTGCTG	AGGGTGTTGATGCGCACGAG	
**HPL**	ACTTCGGCTTCACCATCCTG	GTAGTAGCCCGGCCAGATGA	
**LECTIN**	TCGTCGTCCTTGGAGAGCTT	CATCTGCCAAGTCCCCTTCT	
**LIPASE**	CCAAGAGCCTCATCATCGTG	CGTGGTAGTGGTCCGTGTTG	

**MFS**	CACTGTGGGCTGTGAGCAGT	GCAGGCCGAAATGTCTTGAT	
**CYST2**	TGCCCTGCTCATACTGCTTG	GCGAGTTCCTGGAGGTGAAG	
**PX5**	GGATTGATCCTGCGCTGAG	GACTCGAAGAGGCCCAGGTT	
**CYST**	AGGGCTTGTTCGGTTAGGTG	TGCAGAATAAGGAGCCATGC	
**AOS**	ACCTGTTCACGGGCACCTAC	CGAGGAGCGAGGAGAAGTTG	
**AOC**	CCCCTTCACCAACAAGGTGT	ACCGAGATGTGGCCGTAGTC	
**ACS1**	GATGGTCTCGGATGATCACA	GTCGGGGGAAAACTGAAAAT	
**PR1**	CTGGGTGTCCGAGAAGCAGT	CGGGTTGTAGCTGCAGATGAT	
**PR10**	GTCATGCCGTTCAGCTTCAT	TGTTCTTGCACTCGACTTG	
**PAL**	AAGAAGGTGAACGAGCTGGA	GTTGTCGTTCACGGAGTTGA	
**ACY1**	CGCCGCATGGACTACTTTG	CGACCCGGTGGATACATTTC	
**TPK1**	GATTTGGATTATGGTATAAG	GACACTGCTTATATTTTAGCT	
**TPK2**	GATTCGTCATTGTTTGACCATT	GGAAGAGTCAACATGTTAAGT	
**TMKA**	ACACCGACCATGGAGGACTA	GGTCCAATGCCAGGTCAGAA	

Table S4. The summary of the RNA sequencing and assembly

Samples	Conidiospore			Chlamydospore		
	48th hour (CN1)	72nd hour (CN2)	96th hour (CN3)	48th hour (CH1)	72nd hour (CH2)	96th hour (CH3)
**Total clean reads**	45119630	47962990	42527374	45363344	44547052	41656596
**Total length (bp)**	6767944500	7194448500	6379106100	6804501600	6682057800	6248489400
**Total number of contigs**	11500	11462	11515	11345	11315	11409

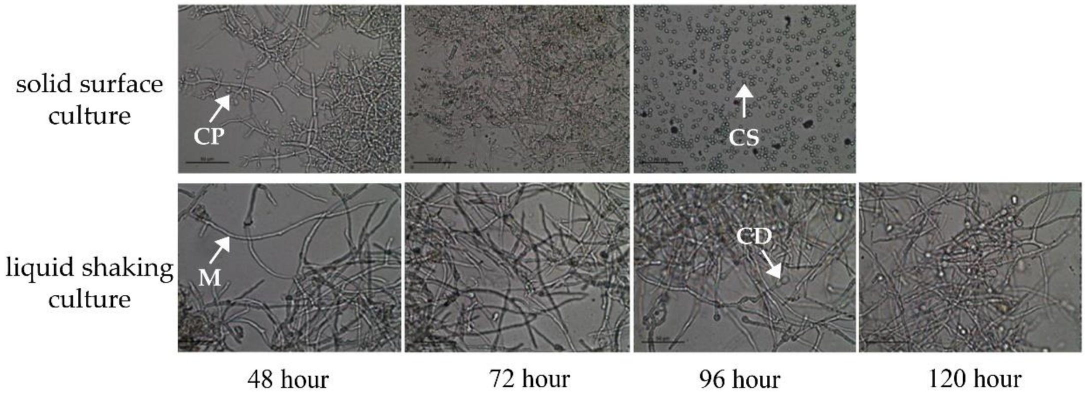

Figure S1. Conidiophores (CP), conidiospores (CS), chlamydospores (CD) and mycelium (M) morphology of *T. asperellum* GDFS 1009 under the light microscope at different time intervals

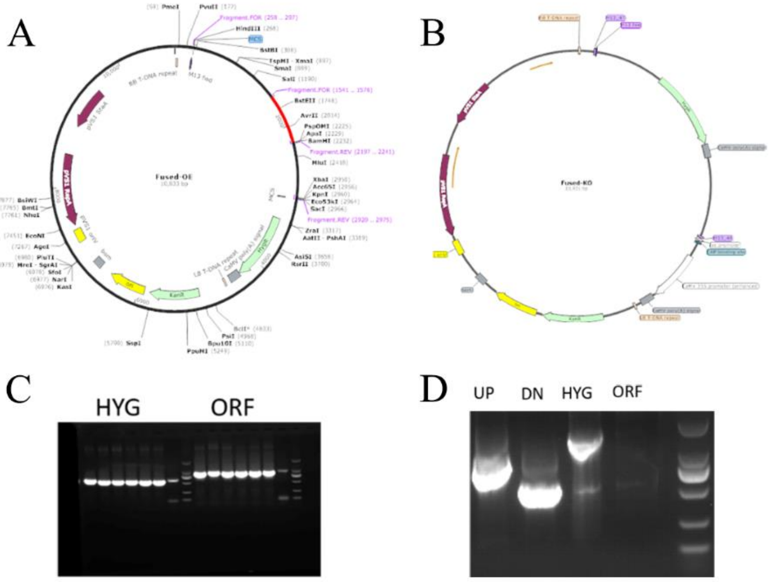

Figure S2. Construction of the Δ*Mup1* and OE*Mup1* mutant strain. A, plasmid map of the *MUP1* overexpression. B, plasmid map of the *MUP1* knockout. C, confirmation of overexpression (presence of hygromycin and mup1 gene). D, confirmation of gene knockout by the presence of hygromycin and absence of MUP1 gene (real-time PCR result of knockout and overexpression strains have been performed to select the best two strains and the results have not been shown).
